# Comparison of Toxicity of CdSe: ZnS Quantum Dots on Male Reproductive System in Different Stages of Development in Mice

**DOI:** 10.22074/ijfs.2015.4610

**Published:** 2015-12-23

**Authors:** Gholamreza Amiri, Akram Valipoor, Kazem Parivar, Mehrdad Modaresi, Ali Noori, Hamideh Gharamaleki, Jafar Taheri, Ali Kazemi

**Affiliations:** 1 Department of Physic, Falavarjan Branch, Islamic Azad University, Isfahan, Iran; 2 Department of Physiology, Basic Science Faculty, Shahrekord Branch, Islamic Azad University, Shahrekord, Iran; 3 Department of Biology, Science and Research Branch, Islamic Azad University, Tehran, Iran; 4 Department of Physiology, Khorasgan Branch, Islamic Azad University, Isfahan, Iran; 5 Department of Biology, Falavarjan Branch, Islamic Azad University, Isfahan, Iran; 6 Department of Biology, Payame Noor University, Tehran, Iran; 7 Department of Chemistry, Islamic Azad University, Shahrekord, Iran

**Keywords:** Quantum Dots, Male Sexual, Development, Toxicity

## Abstract

**Background:**

Quantum dots (QDs) are new types of fluorescent materials for biological labeling. QDs toxicity study is an essential requirement for future clinical applications. Therefore, this study aimed to evaluate cytotoxic effects of CdSe: ZnS QDs on male reproductive system.

**Materials and Methods:**

In this experimental study, the different concentrations of CdSe: ZnS QDs (10, 20 and 40 mg/kg) were injected to 32 male mice (adult group) and 24 pregnant mice (embryo group) on day 8 of gestation. The histological changes of testis and epididymis were studied by a light microscopy, and the number of seminiferous tubules between two groups was compared. One-way analysis of variance (one-way Anova) using the Statistical Package for the Social Sciences (SPSS, SPSS Inc., USA) version 16 were performed for statistical analysis.

**Results:**

In adult group, histological studies of testis tissues showed a high toxicity of CdSe: ZnS in 40 mg/kg dose followed by a decrease in lamina propria; destruction in interstitial tissue; deformation of seminiferous tubules; and a reduction in number of spermatogonia, spermatocytes, and spermatids. However, there was an interesting result in fetal testis development, meaning there was no significant effect on morphology and structure of the seminiferous tubules and number of sperm stem cells. Also histological study of epididymis tissues in both groups (adult and embryo groups) showed no significant effect on morphology and structure of tubule and epithelial cells, but there was a considerable reduction in number of spermatozoa in the lumen of the epididymal duct in 40 mg/kg dose of adult group.

**Conclusion:**

The toxicity of QDs on testicular tissue of the mice embryo and adult are different before and after puberty. Due to lack of research in this field, this study can be an introduction to evaluate the toxicity of QDs on male reproduction system in different stages of development.

## Introduction

Although organic dyes are sensitive to physiological changes and photobleached under normal imaging conditions, they have been widely used for fluorophores imaging and detection of abnormalities. It has been shown that the organic dyes are not applicable for multicolor imaging due to two following properties: i. Presence of signal overlap because of relatively broad emission spectra and ii. Presence of a certain narrow wavelength range in order to be suitably excited (1, 2). However, semiconductor quantum dots (QDs) as tiny light-emitting particles have been applied as new class of fluorescent labels for biology and medicine (3-5).

As compared with organic dyes and fluorescent proteins, inorganic quantum dots have showed an external efficiency of 20-80% quantum, while it is stable under relative harsh environments, with the continuous absorption and the narrow emission spectra (1, 6). Furthermore QDs always emit the same lights according to excitation-emission matrix (EEM), indicating when using one laser execution source, the entire different emission colors from QDs will be observed at the same time. Due to their excellent levels, QDs are also used to determine nucleic acid or protein sequences, so the relative changes in emission intensity are considered as a variant. The long-term multiplexed imaging has recently attracted much attention (2, 7). Therefore, semiconductor QDs are applied for the development of photovoltaic devices (8).

Successful use of QDs has been reported in various medical fields, but the important point is the high toxicity of their core compounds which are composed of heavy metals such as cadmium and thallium (3-5, 9). In recent years, much attention has been paid to the toxic effect of QDs due to its wide use in medical field (10, 11). If it is determined that the combination of heavy metal has a minor role in the cytotoxicity of QDs, there will be a good possibility to limit the use of QDs as contrast agents in clinical applications (5).

Due to lack of *in vivo* studies in this category, this study aimed to evaluate cytotoxic effect of CdSe: ZnS QDs for first time on male reproductive system before and after puberty.

## Materials and Methods

### Method of producing CdSe: ZnS quantum dots

Nanoparticles were synthesized by chemical precipitation method. For this purpose, three solutions of cadmium chloride (CdCl_2_.4H_2_O), mercaptoethanol (ME) and sodium selenite (Na_2_SeO_3_.5H_2_O) were prepared in the distilled deionized water, under vigorous stirring (all chemicals were purchased from Merck Chemical Co., USA). At first, CdCl_2_ solution was poured into a three spout balloon container that was followed by adding ME solution and sodium selenite solution, respectively, to the same balloon under controlled atmospheric condition with nitrogen (N_2_). The resulting solution was mixed with deionized water and centrifuged in order to remove any impurities. Then, the precipitated sample was dried at room temperature. All processes were done at room temperature (12).

The crystal structure and optical properties of QDs were characterized by X-ray diffraction (XRD) pattern using Cu Kα radiation (λ= 0.154 nm) by a Bruker D8 advance XRD machine (Karlsruhe, Germany) and UV-2600 ultraviolet visible spectrophotometer (Shimadzu, Japan). A scanning tunneling microscope (STM, Natsico, Iran) was also used for investigation of particle size distribution.

### Breeding and treatment of animals

In this experimental study, male (n=32) and female (n=24) BALB/c mice weighing 24-30 g with 60-70 days of age were obtained from the Department of Histology, School of Medicine, Shahrekord University of Medical Sciences, Shahrekord, Iran, during 2011-13. The mice were housed in plastic cages and kept for 10 days under 12-hour light/dark conditions, temperature of 22-24˚C, humidity of 50-60%, and free access to food and water in order to adapt their life cycle to new environment. Then, 32 adult male mice were divided randomly into four groups (n=8) as follows: control group and three treatment groups receiving 10, 20 and 40 mg/kg CdSe: ZnS QDs, respectively. In embryo group, 24 female mice were included. The mice were mated and pregnancy was determined by detection of vaginal plug. The pregnant mice were divided randomly into four following groups (n=6): control group and three treatment groups receiving 10 , 20 and 40 mg/kg CdSe: ZnS QDs, respectively. In this study, work with laboratory animals was approved by the Ethics Committee of the Shahrekord University.

### Study design

In adult group, CdSe: ZnS nanoparticles were prepared in normal saline solution and a singledose of 10, 20, and 40 mg/kg was injected intraperitoneally to three treatment groups, respectively. Only saline was injected to the control group. Also in embryo group, CdSe: ZnS nanoparticles were prepared in saline and a single-dose of 10, 20, and 40 mg/kg was injected intraperitoneally to the pregnant mice of three treatment groups on day 8 of gestation, respectively, because the blood-placenta barrier and gonad development begin after days 5 to 7 of gesta tion. Gestation begins with the sign of a vaginal plug as evidence of copulation or gestation day 0.

### Tissue preparing

Ten days after CdSe: ZnS injection, following measurement of body weight, mice were dissected under mild anesthesia, while epididymis and testis organs were rapidly cut, weighted, and immersionfixed in paraformaldehyde. Five micron sections were prepared, dehydrated and embedded in paraffin. The sections were stained using hematoxylin and eosin (H<E) and subsequently processed for histopathological examination under a light microscope. The morphological structure of seminiferous tubules and mean number of spermatogonia, spermatocyte and spermatid were studied in testis. Epithelial height, connective tissue, smooth muscle and sperm density were also studied in epididymis.

### Statistical analysis

Data were analyzed using one-way analysis of variance (one-way ANOVA) by the Statistical Package for the Social Sciences (SPSS, SPSS Inc., USA) version 16. Data were represented as means ± SD. Differences were considered significant at P<0.001.

## Results

### The results of X-ray diffraction and scanning tunneling microscope

The structure of the QDs was investigated by XRD. The sample had a single phase and also a cubic crystal structure. The mean size of the particles was determined by Debye-Scherrer equation that was equal to 2.4 nm for QDs. Also the size was determined around 3 nm using STM (12).

### Histological study of testis in adult and embryo groups

**Fig.1:**
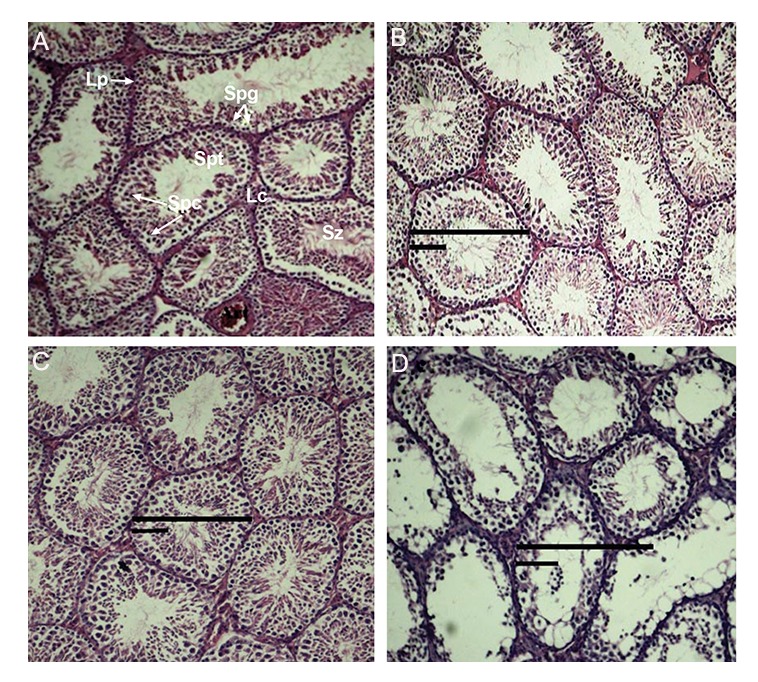
Microscopic images of testis slides of adult group 10 days after injection (H & E, ×400). A-D. Control and treatment groups receiving 10, 20, and 40 mg/kg CdSe: ZnS. Sz; Spermatozoa, Lc; Leydig cells, Lp; Lamina propria, Spg; Spermatogoni, Spc; Spermatocytes and Spt; Spermatids.

**Fig.2:**
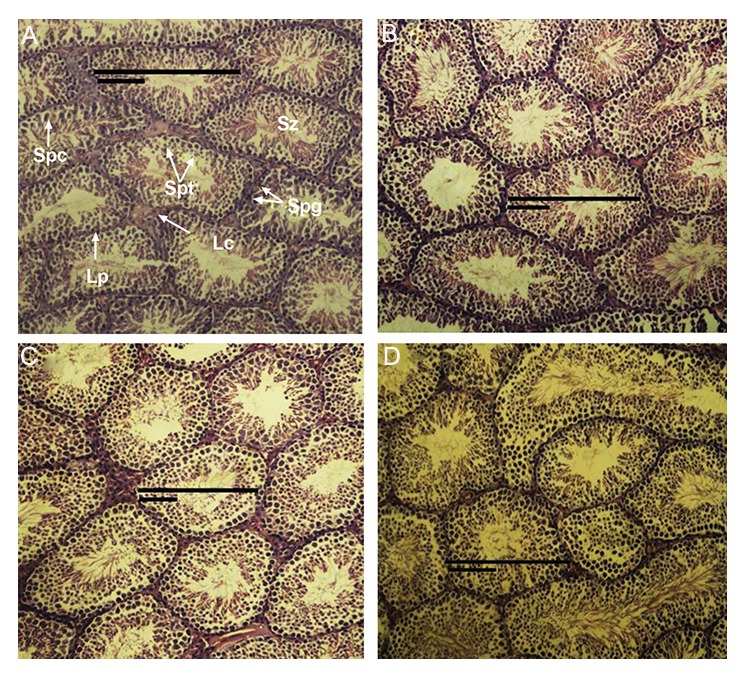
Microscopic images of testis slides of embryo groups (H & E, ×400). A-D. Control and treatment groups receiving 10, 20, and 40 mg/ kg CdSe: ZnS. Sz; Spermatozoa, Lc; Leydig cells, Lp; Lamina propria, Spg; Spermatogoni, Spc; Spermatocytes and Spt; Spermatids.

In adult group, mice of control group and treatment groups receiving 10 and 20 mg/kg CdSe: ZnS QDs showed normal testicular architecture with an orderly arrangement of germinal, and the seminiferous tubules showed normal spermatogenesis pattern, whereas mice of group administered 40 mg/kg CdSe: ZnS QDs showed several tissue alterations of the seminiferous tubules. Testis sections of group given 40 mg/kg CdSe: ZnS QDs depicted moderate to severely damaged seminiferous tubules including the abnormal and disorganization of spermatogenesis cells and destruction of most spermatogenesis’ layers that was clearly recognized in seminiferous tubules. In addition degeneration of the interstitial tissue, blood vessels, widening of the spaces between seminiferous tubules, as well as deformed and atrophic seminiferous tubules were seen (Fig .1). According to histopathology results of testis in adult group, table 1 shows a significant reduction (one-way ANOVA) in mean number of spermatogonia, spermatocytes I and spermatids in group treated with 40 mg/kg CdSe: ZnS QDs. But in embryo group, qualitative studies using an optical microscope showed that morphological structure of seminiferous tubules were similar in treatment and control groups (Fig .2). Also the average numbers of spermatogonia, spermatocytes, spermatids were similar in treatment and control groups (Table 2).

### Histological study of epididymis in adult and embryonic groups

**Fig.3:**
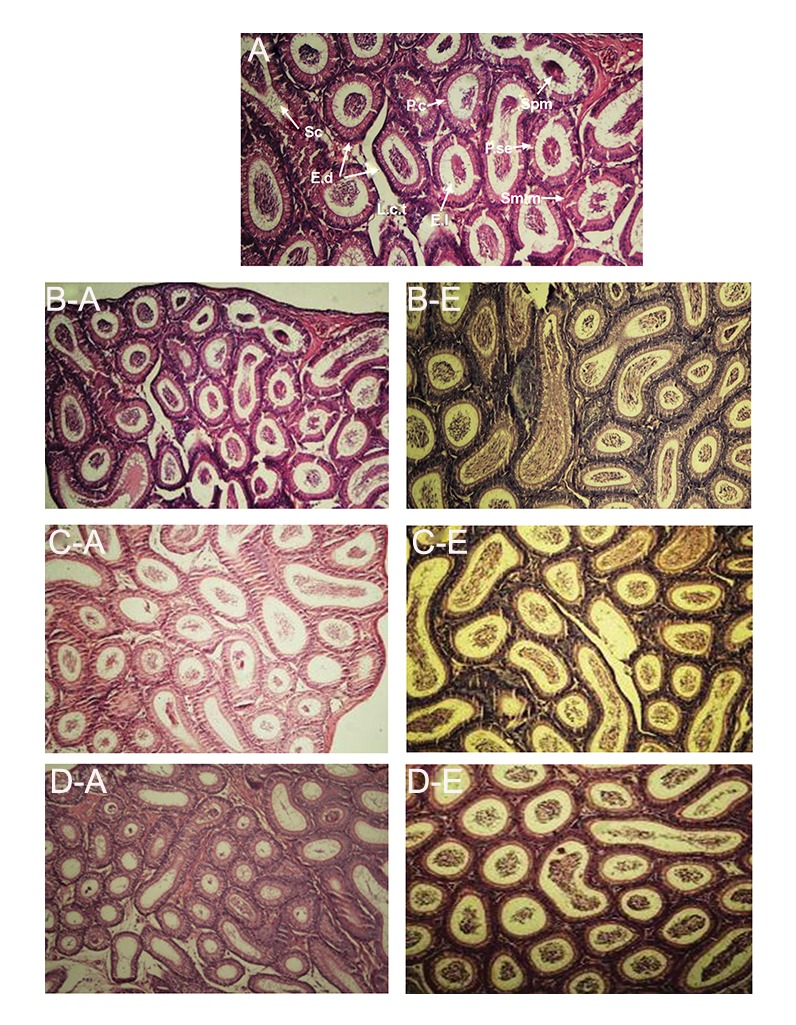
Microscopic images of A. Epididymis in adult and E. Embryo groups after injection of CdSe:ZnS (H & E, ×400), A, B-A, C-A, D-A. Control and treatment groups in adult group and B-E, C-E, D-E. As well as treatment groups in embryo group. L.c.t; Loose connective tissue, E.l; Principal cells, P.se; Pseudostratified stereociliated epithelium, Sm.m; Smooth muscle, Spm; Sperm mass, E.l; Epididymal lumen, E.d; Epididymal duct, Sc; Stereocilia.

Qualitative studies of epididymal tissues using an optical microscope in embryo treatment groups (receiving 10, 20 and 40 mg/kg CdSe: ZnS) and in adult treatment groups (treated with 10 and 20 mg/kg CdSe: ZnS) showed that epididymal epithelium, interstitial tissue and sperm volume in lumen of epididymal duct were similar in treatment and control groups. But in adult group, in the group treated with 40 mg/kg CdSe: ZnS, although epididymal epithelium showed a normal histological appearance, the lumen of epididymal duct was devoid of spermatozoa, indicating the toxic effect of QDs on testis tissue that led to impaired spermatogenesis (Fig .3).

### Body and testis weight changes in adult and embryo groups

In adult group, the testicular weight in the groups treated with 10 and 20 mg/kg CdSe: ZnS QDs were similar to control group and no significant change was found in relative testis weight, but testis weight decreased significantly in mice receiving 40 mg/kg CdSe: ZnS QDs (Fig .2) that was parallel with histological changes in mice testis in this group. The body weight did not change significantly in any of the treatment groups (Table 3). In embryo group, no significant difference was observed in testis weight of treatment groups as compared with the relative value of the control. Also there was no significant difference regarding body weight between the treatment and control groups (Table 4).

**Table 1:** Comparison of mean numbers of sperm in one tubule in adult group after injection


		Groups (n=8 mice)		
Parameter	Control	10 mg/kg	20 mg/kg	40 mg/kg

Spermatogonia	34.55 ± 6.39	33.6 ± 8.94	32.80 ± 6.67	18.85* ± 6.94
Spermatocyte I	44.15 ± 9.35	45.25 ± 6.21	43.80 ± 8.43	29.60* ± 6.86
Spermatid	111.95 ± 33.63	113.65 ± 23.29	109.15 ± 20.72	83.00* ± 23.44


All data are presented as mean ± SD. *; P<0.05.

**Table 2:** Comparison of mean numbers of sperm in one tubule in embryo group


		Groups (n=8 mice)		
Parameter	Control	10 mg/kg	20 mg/kg	40 mg/kg

Spermatogonia	34.15 ± 8.39	34.75 ± 8.96	32.80 ± 9.51	33.90 ± 8.71
Spermatocyte I	44.85 ± 10.55	43.94 ± 7.21	41.10 ± 10.87	44.75 ± 8. 59
Spermatid	111.65 ± 20.01	116.55 ± 14.86	120.90 ± 22.50	110.05 ± 18.77


All data are presented as mean ± SD.

**Table 3:** Comparison of mean values of testis and body weight in adult group 10 days after injection


		Groups (n=8 mice)		
Parameter	Control	10 mg/kg	20 mg/kg	40 mg/kg

Body weight	27.50 ± 1.37	27.00 ± 2.89	29.41 ± 2.20	29.00 ± 1.26
Testis weight	0.093 ± 0.008	0.087 ± 0.012	0.106 ± 0.019	0.055 ± 0.013*


All data are presented as mean ± SD. *; P<0.05.

**Table 4:** Comparison of mean values of testis and body weight in embryo groups


		Groups (n=8 mice)		
Parameter	Control	10 mg/kg	20 mg/kg	40 mg/kg

Body weight	28.60 ± 1.14	26.00 ± 3.52	27.08 ± 2.58	27.25 ± 3.30
Testis weight	0.092 ± 0.010	0.095 ± 0.018	0.090 ± 0.008	0.097 ± 0.025


All data are presented as mean ± SD.

## Discussion

QDs are very effective for long-term fluorescence imaging; however, the potential toxicity of QDs limits their clinical applications. Due to the presence of Cd2+ ions in QDs, they are highly toxic (5, 13). In recent years, cytotoxicity of these particles has been considered highly due to their use in medical field (14). Although the possible toxic effects of nanoparticles on the reproductive system, placenta translocation, and fetus development are still unknown, some researchers have suggested the reproductive toxicity of nanoparticles (15-17). Our study was the first one conducted on toxicity of QDs on the reproductive system. Chan showed that CdSe-core QD induced apoptosis in mouse blastocysts in a dose-dependent manner. Some studied also showed when blastocysts are pretreatment with CdSe-core QD, cell proliferation is inhibited. Furthermore they revealed that CdSe-core QD inhibited post-implantation embryonic development, meaning that they prevented blastocysts to reach the later stages of development as compared to the controls, while the pre-implantation development of morulas into blastocysts was also inhibited by CdSe-core QD. Also CdSe-core QD with concentration of 500 nmol/L caused resorption of post-implantation blastocysts, leading to a decrease in fetal weight. Also the cytotoxicity of CdSe QD in embryonic development was significantly reduced by the addition of a ZnS coating (18). Other studies showed a significant reduction in the rates of oocyte maturation, fertilization, and *in vitro* embryo development that was inducted by the CdSe-core QDs, but there was no reduction when using ZnS-coated CdSe QDs. Treatment of oocytes with CdSe-core QDs with concentration of 500 nM during *in vitro* maturation (IVM) resulted in an increase in resorption of postimplantation embryos and a decrease in placental and fetal weights. It is noteworthy that CdSe-core QDs effectively prevented this cytotoxicity after modification of its surface with ZnS (19).

However, there are some studies regarding toxicity of other nanoparticles on the reproductive system. For example, Yoshida et al. (19) showed C60 (Carbon) nanoparticles administered intratracheally induced adverse effects on the mouse male reproductive function. Also another study showed fetal carbon black nanoparticles (CB-NPs) exposure significantly reduced daily sperm production (DSP) in male offspring. When CB-NPs was administered to adult mice, DSP decreased significantly (20, 21). Furthermore, it was reported that fetal exposure to diesel exhaust (DE) lowered the DSP of male offspring (16). Other researches also indicated that fetal DE exposure may lower DSP in male offspring due to particulate matters in DE, particularly CB. Also in the testis of male offspring, intercellular adhesions of seminiferous epithelium and seminiferous tubules damage were observed (22). In addition *in vitro* studies showed cytotoxic effect of titanium dioxide (TiO_2_) on living power of mice Leydig cells. They also revealed that gold nanoparticles decreased movement of matured sperms, and silver and aluminum nanoparticles were toxic for rat spermatogonia stem cells (21, 22). Also Sleiman et al. (23) showed the impairment in spermatogenesis and a lower sperm count in male Wistar rats that was caused by prepubertal exposure to AgNP. Mathias et al. (24) revealed that Ag nanoparticles reduced the acrosome, plasma membrane integrities, and the mitochondrial activity as well as increased the abnormalities of the sperm. However, there were no changes in sexual behavior, serum hormone concentrations and body growth were. In an experimental study, Ag nanoparticles solution with concentration of 1mg/kg was injected intravenously into male mice over 12 days. No changes were reported in body and testis weights, sperm concentration, motility, fertility indices, follicle-stimulating hormone (FSH) and luteinizing hormone (LH) serum concentrations. However, there were significant changes in serum and intratesticular testosterone concentrations 15 days after initial treatment. Furthermore significant changes in epithelium morphology, germ cell apoptosis and Leydig cell size were observed using a histologic evaluation. Gene expression analysis revealed a significant upregulation in Cyp11a1 and Hsd3b1 in treated animals (25).

In current study, CdSe: ZnS QDs with 2-3 nm size was synthesized by chemical sedimentation method and the cytotoxic effects on male reproductive system was evaluated.. Histopathological studies of testis tissues in adult treatment group receiving 10 and 20 mg/kg CdSe: ZnS and in all embryo treatment groups showed no toxicity. According to our findings, the mean numbers of spermatogonia, spermatocytes, spermatids, as well as matured sperms in seminiferous tubules were similar in above-mentioned treatment groups and control. However, in adult group, our findings revealed that a decrease in testis weight of group receiving 40 mg/kg CdSe: ZnS QDs. Also histological studies of testis tissue showed a high toxicity of CdSe: ZnS in 40 mg/kg dose. Although in this study, cytotoxic effect of CdSe: ZnS QDs on epididymis tissue, testis, and body weight in both adult and embryo groups were studied for the first time, further studies are necessary in this field in order to identify effective background mechanism of QDs cytotoxicity.

## Conclusion

Our findings showed that CdSe: ZnS QDs in dose of 40 mg/kg induced the toxicity in adult mice, although an *in vitro* study has shown that Cd+2 as main reason of QDs toxicity can be effectively prevented by surface modification of CdSe-core QDs with ZnS. It seems that other mechanisms causing QDs toxicity can be detected by quantum dot stability and time between exposure and toxicity. Also comparison of toxicity of the CdSe: ZnS QDs between adults and embryo groups showed that response of organs is different in various development stages, indicating the complicated process of QDs *in vivo* causes their toxicity, in spite of theri obvious advantages in medicine.
